# New insights on Galectin-9 expression in cancer prognosis: An updated systemic review and meta-analysis

**DOI:** 10.1371/journal.pone.0320441

**Published:** 2025-03-26

**Authors:** Chun Yan So, Yusong Li, Kwan Ting Chow

**Affiliations:** Department of Biomedical Sciences, City University of Hong Kong, Kowloon, Hong Kong SAR; Rutgers: Rutgers The State University of New Jersey, UNITED STATES OF AMERICA

## Abstract

Galectin-9 (Gal-9) has gained increasing attention in recent years in the field of cancer immunology. Its interactions with various immune cell types in the tumor microenvironment influence tumor progression, making it a novel target for immunotherapy. Despite its potential as a therapeutic target, the prognostic significance of Gal-9 in tumor cells remains unclear. Conflicting data exists on its expression levels and outcomes, prompting a comprehensive review and meta-analysis to elucidate its independent prognostic role across different cancer types. This study aims to examine the varying effects of Gal-9 expression across various cancer subtypes, providing insights into its potential as a prognostic marker and highlighting its significance in the realm of cancer treatment. To assess the prognostic significance of Gal-9 expression in cancer, we conducted a comprehensive database search across PubMed, Embase, and Web of Science, incorporating studies published until December 2024, regardless of language. Using pooled hazard ratios (HRs) with 95% confidence intervals (CIs), we evaluated the role of Gal-9 expression in predicting cancer outcomes across various cancer types. Our analysis encompassed 29 studies with a total of 4,720 patients to investigate the prognostic significance of Gal-9 expression across different cancer types. The results demonstrated that elevated Gal-9 expression was significantly associated with improved overall survival (OS) in solid tumors, with a pooled hazard ratio of 0.75 (95% CI: 0.63-0.90, p =  0.002). No statistically significant correlation was observed between Gal-9 expression and cancer recurrence (HR =  0.88, 95% CI: 0.65–1.19, p =  0.42). Conversely, in hematological cancers, high Gal-9 expression correlated with more rapid disease progression, as reflected by progression-free survival (PFS) or time to treatment (TTT) (HR =  2.29, 95% CI: 1.26–4.16, p =  0.007). The subgroup analyses further revealed that higher Gal-9 expression was associated with OS in gastrointestinal and urological cancers and was linked to disease-free survival (DFS) and recurrence-free survival (RFS) in hepatobiliary and urological cancers. Our research has uncovered that Gal-9 serves as a promising prognostic indicator for solid tumors, offering valuable insights into patient outcomes. High levels of Gal-9 expression within gastrointestinal, hepatobiliary, and urological cancers have been linked to better prognoses, while its presence in hematological cancers is associated with poorer outcomes. These contrasting findings emphasize the importance of interpreting biomarkers with careful consideration to the specific context. Moreover, our study sheds light on the diverse physiological roles of intracellular and secreted Gal-9, highlighting the intricate ways in which this protein influences cancer progression.

## Introduction

Cancer immunotherapy has significantly transformed cancer treatment in recent decades, with immune checkpoint inhibitors (ICIs) like anti-PD(L)1 and anti-CTLA4 monoclonal antibodies revolutionizing treatment approaches and improving survival rates. Despite their widespread use, treatment responses vary among tumor types, and immune tolerance often develops. Therefore, identifying novel immune checkpoint targets that can enhance anti-cancer immunity is crucial. Galectin-9 (Gal-9), a recently emerging target in cancer immunology, has gained increasing attention as a potential therapeutic agent.

Galectins, a family of carbohydrate-binding proteins, can bind to glycoprotein targets through β-galactose recognition, such as N-acetyl-lactosamine. Fifteen galectins have been identified in humans. Structurally, they feature a conserved carbohydrate recognition domain (CRD) enabling specific carbohydrate ligand recognition. Galectins are categorized into three groups: “Prototype,” “Chimeric,” and “Tandem Repeat.” [1,2]. Gal-9, a member of the “Tandem Repeat” group, has two CRDs connected by a linker region, enabling heterodimer formation and crosslinking with glycoproteins on cell surface receptors, thus modulating cell signaling [3].

Interestingly, Gal-9 exhibits multifaceted roles that are intricately linked to the location of its binding partners at the cellular level. Within the cell, Gal-9 regulates cell adhesion molecules and enhances adhesion, thereby reducing the capacity for tumor invasion and metastasis. Gal-9 has been suggested to impede cancer cell propagation into the extracellular matrix (ECM) and cell adherence to vascular endothelium [4]. Cytosolic Gal-9 also maintains plasma membrane integrity and initiates phagocytosis in immune cells [5]. These findings underscore the function of Gal-9 as a critical player in tumor suppression at the cellular level.

Conversely, extracellular Gal-9 serves a completely different role, particularly within the tumor microenvironment (TME). The TME consists of both malignant and normal cell populations, including a diverse array of infiltrating immune cell types, as well as stromal tissue components. Communication between the various cellular constituents of the TME is facilitated by a complex network of secreted signaling molecules including cytokines, chemokines, growth factors and enzymes [6]. Extracellular Gal-9 interacts with surface receptors on immune cells in the TME. On the T cell surface, Gal-9 acts as a ligand for multiple receptors, including TIM-3, PD-1, VISTA, and CD40 [7–9]. These interactions negatively impact the secretion of IFN-γ, leading to T cell exhaustion and apoptosis [10]. Moreover, Gal-9 enhances the differentiation and stability of regulatory T cells (Tregs) through interactions with CD44 and DR3, promoting an anti-inflammatory response [9,11]. In macrophages, Gal-9 binding to the innate receptor dectin-1 promotes M2 polarization, which in turn suppresses anti-tumor immune responses [12]. In natural killer (NK) cells, Gal-9 has been shown to stimulate interleukin (IL)-10 production, which is a potent immunosuppressive cytokine leading to immune escape [13]. Extracellular Gal-9 secreted by cancer cells are also shown to promote myeloid derived suppressor cells (MDSCs) via suppression of stimulator of interferon genes (STING) pathway [14]. Within monocytes, intracellular Gal-9 is found to be responsible for activating inflammatory cytokine genes [15]. Furthermore, in patients with virus-associated solid tumors, elevated cell surface expression of Gal-9 on NK cells correlates with reduced cytotoxicity due to impaired production of perforin and granzyme B [16]. These lines of evidence demonstrate that Gal-9 negatively impacts anti-tumor immunity.

The prognostic significance of Gal-9 in human cancers is not well-defined. Available evidence suggests that Gal-9’s effect is highly context-dependent, acting as a “double-edged sword”. Differences in the physiological roles of intracellular versus extracellular Gal-9 make it a compelling immune checkpoint target for further investigation. A previous meta-analysis [17] in 2018 suggested that Gal-9 overexpression in solid tumors, particularly gastrointestinal cancers, may be associated with improved survival. However, conflicting results and a surge in recent publications highlight the need for an updated meta-analysis. This study aims to consolidate and integrate existing literature to uncover the prognostic impact of Gal-9 across various cancer subtypes. This comprehensive assessment will provide deeper insights into the clinical and therapeutic relevance of this immune checkpoint molecule and its association with cancer recurrence and survival.

## Materials and Methods

The methodology for this systematic review adheres to the PRISMA 2020 guidelines (S2 File) and is registered with PROSPERO (CRD42024543015).

### Literature search strategy

We conducted an extensive search in PubMed, Embase, and Web of Science for articles published up to December 2024, regardless of language or region, using the following searching strategies and keywords:

(Galectin9) OR (lectin, galactoside-binding, soluble, 9, human) OR (galectin-9, human) OR (ecalectin, human) OR (lectin, galactoside-binding, soluble, 9 (galectin 9), human) OR (galectin 9, human) AND (Cancer) OR (Tumor) OR (Neoplasm) OR (Tumors) OR (Neoplasia) OR (Neoplasias) OR (Cancers) OR (Malignant Neoplasm) OR (Malignancy) OR (Malignancies) OR (Malignant Neoplasms) OR (Neoplasm, Malignant) OR (Neoplasms, Malignant) AND (prognosis) OR (Prognoses) OR (Prognostic Factors) OR (Prognostic Factor) OR (Factor, Prognostic) OR (Factors, Prognostic)

### Eligibility

Studies were included only if they met ALL the following criteria:

1. Original research articles published as full-length studies.
2. Examined Gal-9 expression in histological samples or patient plasma and correlated it with survival outcomes.
3. Provided sufficient data, including hazard ratios (HR) with 95% confidence intervals (CI), either directly reported or estimated, to enable prognostic analysis.

Studies that did not meet ALL the above criteria or with missing clinical data were excluded from the analysis.

### Data collection and quality assessment

The reviewed studies underwent a thorough analysis by two authors utilizing a standardized approach to gather essential details. This information encompassed publication year, primary author, study location, sample size, research methodology, cancer subtype, specimen used, duration of follow-up, methodology for Gal-9 detection and expression analysis, statistical models employed, and clinical findings. In cases where a study reported both univariate and multivariate hazard ratios, preference was given to the latter to consider potential confounders. The quality of the studies was meticulously assessed using the Newcastle-Ottawa Scale (NOS) criteria. Both authors individually assessed the NOS scores for each study and ultimately reached a consensus through deliberation. The evaluation process adhered to the Reporting Recommendations for Tumor Marker Prognostic Studies (REMARK) guidelines to ascertain robust methodology and reporting standards. Any discrepancies between the two authors during data collection and quality assessment were resolved through consultation with a third author to ensure accuracy and reliability in the analysis.

### Statistical analyses

Our meta-analysis focused on assessing overall survival (OS), cancer-specific survival (CSS), disease-free survival (DFS), and recurrence-free survival (RFS), as they all represent crucial clinical outcomes. The analysis treated these variables as a collective parameter for evaluation purposes. Utilizing Review Manager 5.4 and Stata 18.0 software, data analyses were conducted to determine the prognostic significance of Gal-9 in various solid tumors. Outcome measures included OS/CSS and DFS/RFS. The degree of heterogeneity among studies was assessed using the I^2^ statistic. A value exceeding 50% indicated substantial heterogeneity, which required the application of a random-effects model. Conversely, a fixed-effects model was employed when the I^2^ value was 50% or less. Each study’s weight was determined based on its contribution to the pooled estimate, calculated by taking the inverse of the variance of the effect, which is closely related to its sample size.

To evaluate the influence of each study on the overall findings, a sensitivity analysis was performed by excluding one study at a time. Various tests including funnel plots, Begg’s test, and Egger’s test were utilized to detect publication bias effectively. The level of significance of p <  0.05 was applied for statistical analyses. This rigorous approach ensured a comprehensive assessment of the data and its implications.

## Results

### Study selection

**Fig 1:**
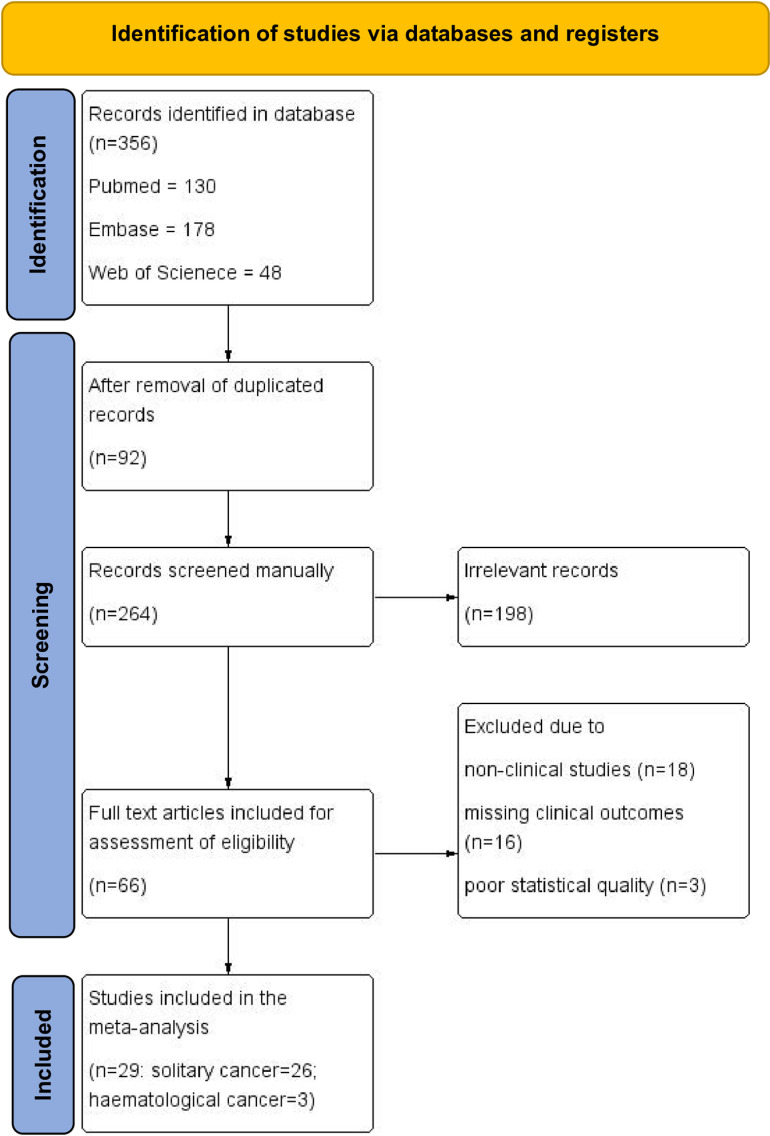
PRISMA flow diagram.

Our extensive review of existing literature began with 356 studies that were scoured for relevance. After removing 92 duplicated entries and with careful evaluation of titles and abstracts in the 264 records, 198 studies were deemed unfitting for the topic and were subsequently excluded. The remaining 66 studies underwent a more rigorous assessment, leading to the inclusion of 29 studies in our meta-analysis. Of these, 26 studies focused on solid cancers while the other 3 centered on hematological cancers. Of the excluded studies, 18 were eliminated for a lack of clinical significance, 16 for missing crucial clinical outcomes, and 3 for poor statistical quality, calling into question the reliability and consistency of their data. Summary of all the studies identified in literature search was listed in (S1 File). The selection process was described by a PRISMA flow diagram (Fig 1).

### Demographics and clinical characteristics

The meta-analysis incorporated data from 29 studies published between 2013 and 2024, involving a total of 4,720 patients. These studies were conducted across 9 countries: 16 in Asian countries, 8 in European countries, 4 in North American countries, and 1 in an African country. The study designs comprised 20 retrospective cohorts and 9 prospective cohorts.

Regarding cancer subtypes, among the 26 studies on solid tumors, the majority focused on hepatobiliary cancers (n = 6, including 5 liver cancers and 1 pancreatic/ampullary cancer), followed by gastrointestinal cancers (n = 5, including 3 gastric cancers, 1 colon cancer and 1 esophageal cancer), gynecological cancers (n = 4, including 2 ovarian cancers, 1 uterine cancer and 1 cervical cancer), lung cancers (n = 4, including 3 non-small cell lung cancer and 1 small cell lung cancer), urological cancers (n = 3, including 2 kidney cancers and 1 bladder cancer). Additionally, there were 2 studies on breast cancer, 1 study on brain cancer, and 1 study on melanoma. The 3 hematological cancer studies all focused on chronic lymphocytic leukemia.

Most of the solid tumor studies used immunohistochemical staining (IHC) and immunofluorescence (IF) methods to detect and localize Gal-9 intracellularly (in the cytoplasm or membrane) in tumor cells. For hematological cancers, enzyme-linked immunosorbent assay (ELISA) or quantitative polymerase chain reaction (qPCR) were used to detect plasma Gal-9 level in the circulating bloodstream. Various cutoff values and methods for Gal-9 assessment were employed across the different studies, where the most frequently used cutoff was based on a predefined IHC intensity or the median expression.

For the clinical outcomes analysis, pooled overall survival (OS), cancer-specific survival (CSS), disease-free survival (DFS) or recurrence-free survival (RFS) were calculated using a Cox regression model, majority being a multivariate model. For hematological cancers, pooled progression-free survival (PFS) from 3 studies were obtained, with 2 studies using a multivariate model and 1 using a univariate model. Results of the survival data from all studies were summarized in (S1 Table). Most of the included studies had reasonably long follow-up durations, up to 10 years. All the included studies scored above 5 on the Newcastle-Ottawa Scale (NOS), indicating reasonably high methodological quality (Tables 1 and 2).

**Table 1:** Summary of the included studies.

Author	Year	Origin of study	No. of cases	Study design	Cancer type	Specimen	Gal-9 detection method	Localization	Cut-off value (for high Gal-9)	Follow-up duration	Outcomes measured	Cox model	High-Gal9	Low-Gal9
**Solid tumors**														
Knudsen [18]	2021	Denmark	163	RC	Brain (GBM)	FFPE	IHC and IF	Tumor	median	up to 10 years	OS	multi	82	81
Yoshikawa [19]	2022	Japan	62	RC	Breast (TNBC)	FFPE	IHC	Tumor	>=1% of tumor cells	median = 58 months	RFS	multi	49	13
Melief [20]	2017	Netherlands	73	PC	Melanoma	FFPE	IHC and IF	Tumor and immune cells	median	up to 10 years	OS	multi	36	37
Wang [21]	2016	China	90	PC	Colon	FFPE	IHC	Tumor	IHC score>=2	up to 8 years	OS	multi	51	39
Choi [22]	2017	Korea	619	PC	Gastric	TMA	IHC	Tumor	>=10% of tumor cells	median = 65.7 months	OS	multi	327	292
Hou [23]	2017	China	45	RC	Esophageal	FFPE	IHC	Tumor	H-score>=3	N.R.	OS	multi	7	38
Jiang [24]	2013	China	183	PC	Gastric	TMA	IHC	Tumor	H-score > 200	median = 40 months	OS	multi	62	121
Wang [25]	2018	China	587	RC	Gastric	TMA	IHC	Tumor	median	median = 48 months	OS	multi	293	293
Fu [26]	2015	China	196	RC	Kidney	TMA	IHC	Tumor	median	median = 106 months	OS/RFS	multi	89	107
Jikuya [27]	2020	Japan	83	RC	Kidney	TMA	IHC	Tumor	median	2 to 10 years	OS/RFS	multi for OS, uni for RFS	24	59
Liu [28]	2017	China	202	RC	Bladder	TMA	IHC	Tumor	minimum P value method	median = 60.5 months	RFS/CSS	multi	102	100
Gu [29]	2013	China	147	PC	Liver	TMA	IHC	Tumor	N.R.	median = 24.4 months	OS/RFS	multi	68	79
Jiao [30]	2022	China	140	RC	Liver	FFPE	IHC and IF	Tumor	IHC score > 0	median = 51 months	OS	multi	93	47
Kong [31]	2020	China	247	RC	Liver	TMA	IHC	Tumor	ROC curve for survival	median = 60 months	OS	uni	109	138
Sideras [32]	2017	US	224	RC	Pancreas and ampullary	TMA	IHC	Tumor	Lowest -2log likelihood	up to 10 years	OS	multi	120	87
Sideras [33]^]^	2019	US	31	RC	Liver	TMA and serum	IHC and ELISA	Tumor/Plasma	Lowest -2log likelihood	up to 10 years	OS	multi	20	11
Zhang [34]	2012	China	200	RC	Liver	FFPE	IHC	Tumor	IHC score>=3	up to 10 years	CSS	multi	113	87
Chen [35]	2020	China	102	RC	Lung (SCLC)	FFPE	IHC	TIL	>=30% staining	2 to 6 years	RFS	multi	74	28
He [36]	2019	Poland	136	RC	Lung (NSCLC)	FFPE	IHC	Tumor	>=30% staining	N.R.	OS/RFS	multi for RFS, uni for OS	9	127
Schulkens [37]	2014	Netherlands	87	RC	Lung (NSCLC)	FFPE	qPCR	Frozen tissue	Median mRNA exp level	Up to 5 years	OS/RFS	uni	43	44
Beyer [38]	2022	Germany	83	RC	Cervix	TMA	IHC	Tumor	IRS>=1	median = 100 months	OS/RFS	multi	41	42
Labrie [39]	2017	Canada	186	RC	Ovary	TMA	IF/ELISA	Tumor/Plasma	H-score > 3	up to 10 years	OS	multi	98	88
Schulz [40]	2018	Germany	147	RC	Ovary	FFPE	IHC	Tumor	IRS > 6	up to 10 years	OS	multi	36	111
Beyer [41]	2024	Germany	225	RC	Uterus	FFPE	IHC	Tumor	IRS>=3	more than 10 years	OS/RFS	multi	N.R.	N.R.
Grosset [42]	2016	Canada	98	RC	Breast	FFPE	IHC	Tumor	IHC score>=6	N.R.	RFS	uni	45	53
Ohue [43]	2016	Japan	120	PC	Lung (NSCLC)	TMA	IHC	Tumor	IHC score>=3	N.R.	OS	multi	37	83
**Hematological cancers**														
Ahmed [44]	2024	Egypt	91	PC	CLL	serum	ELISA	Plasma	median	up to 4 years	PFS	multi	47	44
Alimu [45]	2023	China	53	PC	CLL	serum	ELISA	Plasma	ROC	N.R.	PFS	uni	NR	NR
Bojarska-Junak [46]	2023	Poland	100	PC	CLL	serum	qPCR	Circulating mRNA	ROC	median = 52 months	TTT	multi	48	52

Summary of all the studies included

List of abbreviations in Table 1:

RC: retrospective cohort, PC: prospective cohort, GBM: Glioblastoma Multiforme, TNBC: Triple negative breast cancer, SCLC: Small cell lung cancer, NSCLC: Non-small cell lung cancer, CLL: Chronic lymphocytic leukemia, FFPE: Formalin fixed and paraffin embedded, TMA: Tissue microarray, IHC:

Immunohistochemistry, IF: Immunofluorescence, ELISA: Enzyme-linked immunosorbent assay, qPCR: Quantitative polymerase chain reaction, TIL: Tumor infiltrating lymphocytes, ROC: Receiver operating curve, IRS: Immuno-reactivity score, H-score: Histochemical score, N.R.: Not reported.

**Table 2:** Newcastle-Ottawa Scale (NOS) of the selected studies.

Study ID	Selection				Comparability		Outcome			Total
	Representativeness of the exposed cohort	Selection of the non-exposed cohort	Ascertainment of exposure	Outcome of interest was not present at start of study	Comparability of cohorts on the basis of the design	Comparability of cohorts on the basis of the analysis	Assessment of outcome	Follow-up long enough for outcomes to occur	Adequacy of follow up of cohorts	
Knudsen 2021	*	*	*	*	*	*	*	*	*	9
Yoshikawa 2022	*	*	*	*	0	*	*	*	*	8
Melief 2017	*	*	*	*	*	*	*	*	*	9
Wang 2016	*	*	*	*	*	*	*	*	*	9
Choi 2017	*	*	*	*	*	*	*	*	*	9
Hou 2017	*	*	*	*	0	*	*	0	0	6
Jiang 2013	*	*	*	*	*	*	*	*	*	9
Wang 2018	*	*	*	*	*	*	*	*	*	9
Fu 2015	*	*	*	*	*	*	*	*	*	9
Jikuya 2020	*	*	*	*	*	*	*	0	0	7
Liu 2017	*	*	*	*	*	*	*	*	*	9
Gu 2013	*	*	0	*	*	*	*	*	*	8
Jiao 2022	*	*	*	*	*	*	*	*	*	9
Kong 2020	*	*	*	*	*	0	*	*	*	8
Sideras 2017	*	*	*	*	*	*	*	*	*	9
Sideras 2019	*	*	*	*	0	0	*	*	*	7
Zhang 2012	*	*	*	*	*	*	*	*	*	9
Chen 2020	*	*	*	*	*	*	*	*	*	9
He 2019	0	*	*	*	0	*	*	0	0	5
Schulkens 2014	*	*	*	*	0	0	*	*	*	7
Beyer 2022	*	*	*	*	*	*	*	*	*	9
Labrie 2017	*	*	*	*	*	*	*	*	*	9
Schulz 2018	*	*	*	*	*	*	*	*	*	9
Beyer 2024	*	*	*	*	*	*	*	*	*	9
Grosset 2016	*	*	*	*	*	0	*	0	0	6
Ohue 2016	*	*	*	*	*	*	*	0	0	7
Ahmed 2024	*	*	*	*	*	*	*	*	*	9
Alimu 2023	*	*	*	*	0	0	*	0	0	5
Bojarska-Junak 2023	*	*	*	*	*	*	*	*	*	9

### Galectin-9 expression and overall survival in solid tumors

**Fig 2:**
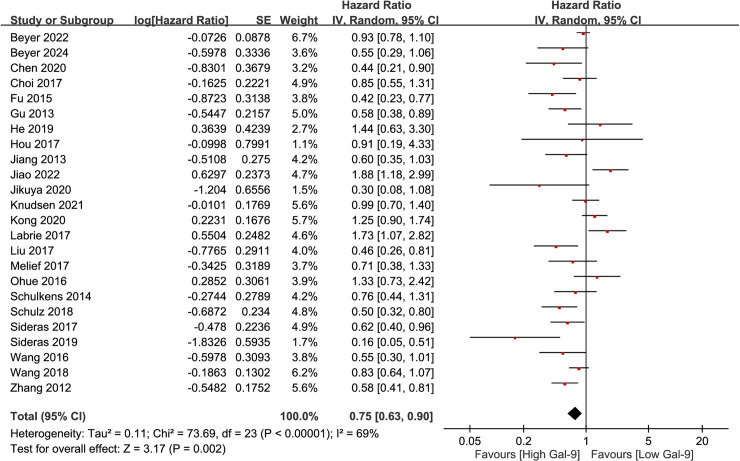
Gal-9 expression and OS/CSS in solid tumors.

We first focused on Gal-9 expression in solid tumors. A review of 24 studies involving 4012 patients were analyzed for OS and CSS. The findings indicated a positive correlation between high Gal-9 expression in solid tumors and improved OS or CSS outcomes (HR =  0.75, 95% CI =  0.63-0.90, p =  0.002). The analysis utilized a random-effects model due to notable heterogeneity among the studies (I^2^ =  69%, p < 0.00001) (Fig 2).

**Fig 3:**
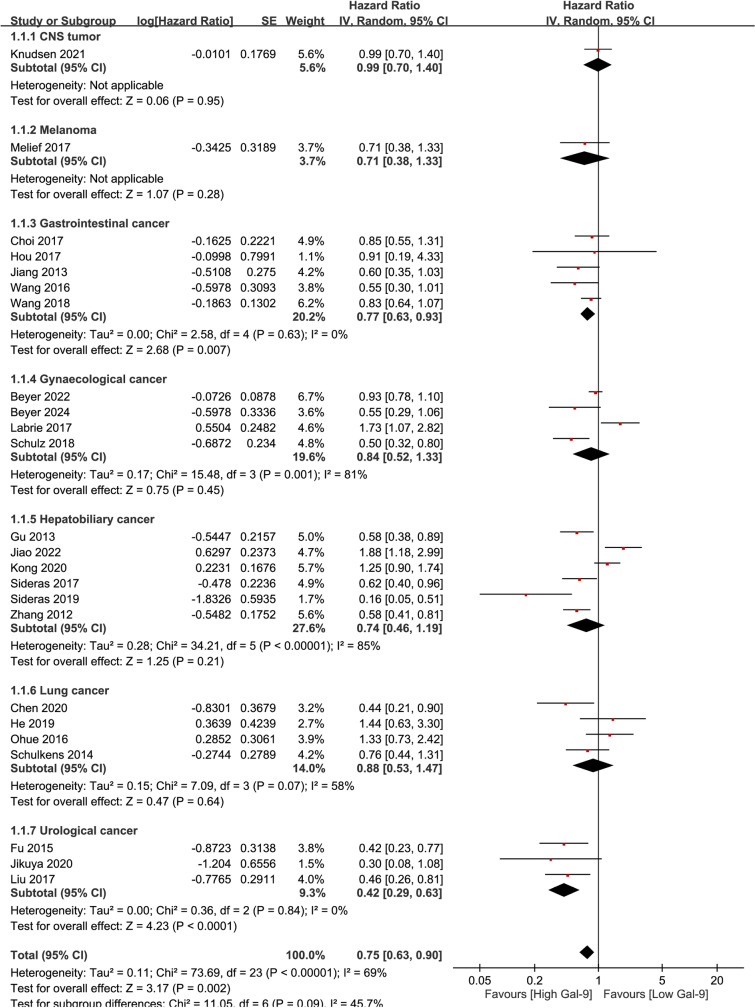
Subgroup analysis on OS/CSS in solid tumors.

Subgroup analyses were conducted to investigate the relationship between increased Gal-9 expression and patient outcomes in different cancer subtypes. The results revealed a significant correlation between higher Gal-9 levels and improved OS in patients with gastrointestinal cancers, including esophageal and gastric cancers (HR = 0.77, 95% CI = 0.63-0.93, p = 0.007). There was low heterogeneity observed in this subgroup analysis (I^2^ = 0.00%, p = 0.63). Furthermore, urological cancers, including kidney and bladder cancers, also exhibited a significant association between elevated Gal-9 expression and longer OS (pooled HR = 0.42, 95% CI = 0.29-0.63, p < 0.0001), with minimal heterogeneity detected (I^2^ = 0.00%, p = 0.84). In contrast, there were no statistically significant associations found for gynecological cancers (HR =  0.84, 95% CI =  0.52–1.33, p =  0.45), hepatobiliary cancers (HR =  0.74, 95% CI =  0.46–1.19, p =  0.21), or lung cancer (HR =  0.88, 95% CI =  0.53–1.47, p =  0.64) (Fig 3).

### Galectin-9 expression and disease-free survival in solid tumors

**Fig 4:**
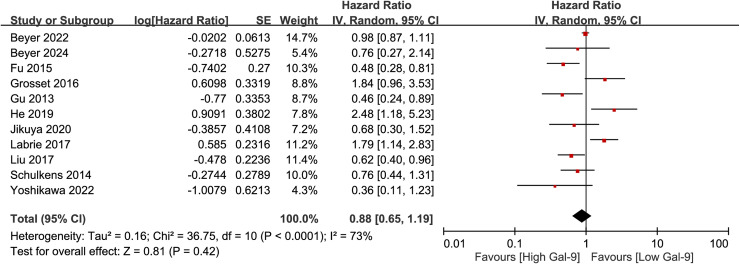
Gal-9 expression and DFS/RFS in solid tumors.

The meta-analysis included 11 studies that looked at hazard ratios for DFS or RFS. Unlike findings for OS/CSS, there was no significant correlation between Gal-9 expression levels and DFS/RFS when all solid tumors were analyzed as a group (HR =  0.88, 95% CI =  0.65–1.19, p =  0.42) (I^2^ =  73%, p <  0.0001) (Fig 4).

**Fig 5:**
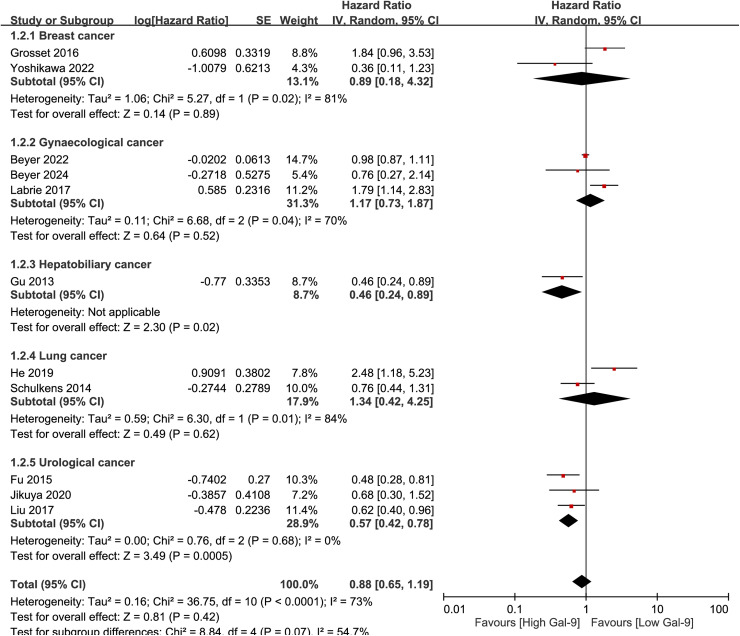
Subgroup analysis on DFS/RFS in solid tumors.

In contrast, subgroup analysis by cancer types revealed significant positive correlation with DFS in urological cancers (HR =  0.57, 95% CI =  0.42-0.78, p = 0.0005) (I^2^ =  0.00%, p =  0.68). However, for other cancer types, including gastrointestinal, gynecological, and lung cancers, there was no significant association between Gal-9 expression and DFS/RFS. The only study for hepatobiliary cancer (HR = 0.46, 95% CI =  0.24-0.89, p = 0.02) showed significant correlation with DFS (Fig 5).

To further investigate the causes of heterogeneity other than cancer subtypes, we further performed subgroup analysis (Table 3). We found that retrospective studies and studies with non-IHC Gal-9 detection methods have more heterogeneous outcomes in both OS and DFS. Moreover, high Gal-9 expression in Asian studies tends to have better prognosis but not in European or North American studies.

**Table 3:** Further subgroup analysis of OS/CSS and DFS/RFS.

Subgroup	OS/CSS			
	No. of study	HR (95%CI)	*P*	*I* ^2^
**Study design**				
RC	18	0.76 (0.61-0.94)	<0.00001	74%
PC	6	0.72 (0.56-0.93)	0.23	27%
**Gal-9 detection method**				
IHC or IF	21	0.75 (0.63-0.89)	<0.0001	65%
ELISA or qPCR	3	0.68 (0.23-1.96)	0.0004	87%
**Region**				
Asian	14	0.73 (0.57-0.93)	<0.0001	70%
European	7	0.81 (0.65-1.01)	0.11	43%
North American	3	0.63 (0.22-1.81)	0.0001	89%
Subgroup	DFS/RFS			
	No. of study	HR (95%CI)	*P*	*I* ^2^
**Study design**				
RC	10	0.94 (0.69-1.28)	0.0002	72%
PC	1	0.46 (0.24-0.89)	N.A.	N.A.
**Gal-9 detection method**				
IHC or IF	9	0.81 (0.58-1.15)	0.0004	72%
ELISA or qPCR	2	1.18 (0.51-2.75)	0.02	82%
**Region**				
Asian	5	0.54 (0.41-0.71)	0.82	0%
European	4	1.06 (0.70-1.58)	0.07	57%
North American	2	1.81 (1.25-2.63)	0.95	0%

N.A.: Not available as only one study included

### Galectin-9 expression and progression free survival in hematological cancers

**Fig 6:**
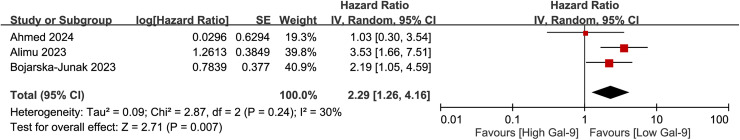
Gal-9 expression and PFS/TTT in hematological cancers.

Interestingly, for hematological cancers (all of which are chronic lymphocytic leukemia studies), higher Gal-9 expression was associated with shorter PFS and time to treatment (TTT), indicating rapid disease progression (HR =  2.29, 95% CI =  1.26-4.16, p = 0.007) (I^2^ =  30%, p =  0.24). These findings highlight the potential impact of Gal-9 in predicting disease progression in this patient population (Fig 6).

### Publication bias assessment

The potential for publication bias in our meta-analysis was carefully evaluated using a combination of funnel plots and Egger’s tests. Visual inspection of the funnel plots revealed no significant asymmetry. Furthermore, the results of Egger’s tests for OS/CSS in solid tumors (P =  0.097), DFS/RFS in solid tumors (P =  0.773), and PFS/TTT in hematological cancers (P =  0.376) all indicated the absence of publication bias (S1–3 Figs).

### Sensitivity analysis

A sensitivity analysis was performed via systematically excluding individual studies using a random-effects model. The findings indicated that the overall outcome trends remained stable even when specific studies were removed. This was observed across various parameters for solid tumors, including OS/CSS and DFS/RFS (S4-5 Figs).

However, for hematological cancer, the situation was different due to the small number of studies included (n = 3). Specifically, the removal of the study by Bojarska-Junak et al. led to a significant alteration of the overall result since this study included the greatest number of patients among the hematological cancer studies (S6 Fig).

The sensitivity analysis suggests that the meta-analysis results for solid tumors are robust and not heavily influenced by any individual study. For hematological cancers, the small number of studies and the disproportionate influence of the largest study indicate the need for caution in interpreting the overall result.

## Discussion

Our study demonstrated that high Gal-9 expression in solid tumors is generally associated with a more favorable prognosis compared to low Gal-9 expression. This association was statistically significant in the pooled meta-analysis for overall survival (OS) and cancer-specific survival (CSS) outcomes in solid tumors, but not for disease-free survival (DFS) and recurrence-free survival (RFS), except for urological cancers. In hematological cancers, the opposite was observed, where high Gal-9 expression was associated with an inferior prognosis in terms of progression-free survival (PFS). It should be noted that all studies investigating Gal-9 expression in hematological cancers were conducted using plasma samples, indicating that the measured Gal-9 was primarily extracellular [39,40]. In contrast, only 2 out of the 26 studies for solid tumors attempted the detection of extracellular Gal-9 using ELISA, while the rest mainly focused on intracellular expression using immunohistochemical methods or qPCR. Further subgroup analysis showed that high Gal-9 expression in solid tumors has been consistently linked to improved prognosis in Asian studies, a trend not observed in European or North American populations. The differences may be primarily attributed to the exclusive use of IHC in Asian studies, which captures only intracellular Gal-9 expression. Also, significant variability in patient characteristics and Gal-9 cutoff values across Western studies introduces heterogeneity that may obscure the prognostic implications of Gal-9. Additionally, gender, ethnic diversity and genetic variations influencing Gal-9 expression could play a potential role in these differences. Additional studies are required to pinpoint the parameters underlying this discrepancy.

It is important to note that most studies examining Gal-9 expression in solid tumors have utilized direct antigen detection methods, such as IHC or IF staining, which primarily capture the cytosolic pool of Gal-9. Noteworthy, among the included studies on ovarian cancer in our meta-analysis, two of them showed diverse outcomes. Labrie et al. [39] demonstrated inferior DFS in high-grade serous ovarian carcinoma with high plasma Gal-9, while Schulz et al. [40] reported the opposite using IHC to detect Gal-9 expression in formalin-fixed paraffin-embedded (FFPE) samples. These findings suggest that the release of Gal-9 from tumor cells is not only an important biomarker but may also play a critical role in mediating the function of other cells, including immune cells.

The functions of extracellular Gal-9 are quite diverse depending on the target cell types. Unlike most secreted proteins that utilize the conventional endoplasmic reticulum (ER) pathway for exocytosis, Gal-9 lacks the signal peptide sequence necessary for transport into the ER [47]. As a result, the precise mechanism of Gal-9 excretion remains poorly understood. Possible pathways for Gal-9 release include direct translocation across the plasma membrane, as well as packaging and release via extracellular vesicles [48]. In cell types such as T and NK cells, Gal-9 has been found to be expressed on the cell surface, which correlated with functional exhaustion [16]. In solid tumors, it is postulated that TGF-β expression, driven by HIF-1 in response to tumor hypoxia, could initiate an autocrine pathway that leads to increased secretion of Gal-9. This TGF-β-rich TME is unfavorable for T cell proliferation and maturation [49,50]. In pancreatic cancer, serum Gal-9 is elevated in patients with pancreatic adenocarcinoma compared to healthy individuals with benign pancreatic lesions [51]. For hematological cancers, Gal-9 is released readily in the blood stream. In acute myeloid leukemia (AML) cells, TIM-3 expressed on the cell surface participates in Gal-9 secretion, releasing it in a free soluble form in the plasma [52]. Elevation of plasma Gal-9 is linked to treatment resistance and poor prognosis. Furthermore, it is reported that the interaction between Gal-9 and TIM-3 plays a crucial role in sustaining leukemic stem cells and promotes immune evasion in AML models [53]. These lines of evidence suggest that elevated serum Gal-9 is correlated with disease severity and prognosis.

Adding to the complexity, the expression of Gal-9 in tumor cells and in tumor-infiltrating lymphocytes (TILs) appears to have different functional consequences. This is evident from the divergent prognostic implications in these two cellular compartments in non-small cell lung cancer (NSCLC): High Gal-9 expression on the tumor cells is correlated with improved OS in NSCLC patients [35]. In contrast, high Gal-9 expression on the TILs is associated with decreased RFS in NSCLC [36]. This phenomenon is also evident in other cancer subtypes including ovarian cancer [39] and glioma [54]. This may be explained by the fact that Gal-9 released from tumor cells suppresses anti-tumor immunity by interacting with various surface receptors including TIM-3, PD-1, VISTA, CD40, CD44 and DR3 [7–12] in T lymphocytes, leading to T cell exhaustions and immune tolerance.

Conversely, when Gal-9 is expressed solely in tumor cells and not released, the functional outcomes differ significantly. Rather than interacting with immune cells, it is found that the expression of Gal-9 on the surface of tumor cells reduces the invasiveness of breast cancer cells by limiting their capacity to invade the surrounding extracellular matrix (ECM) and attach to the vascular endothelium [55]. In murine models of melanoma and colon cancer, Gal-9 has been shown to suppress tumor metastasis by inhibiting the extravasation of circulating tumor cells and their adhesion to the ECM. Furthermore, Gal-9 demonstrates an anti-neoplastic effect on colon and esophageal cancer *in vitro* by inducing apoptosis and inhibiting angiogenesis [56,57]. The favorable prognosis associated with high intracellular Gal-9 expression can be explained by its role in enhancing cell-cell aggregation. Consequently, increased intracellular Gal-9 expression in cancer cells is associated with reduced metastatic potential, which may account for the improved overall survival OS and RFS outcomes observed in patients with solid malignancy, in particular gastrointestinal cancers.

One of the strengths of our study is its comprehensive and up-to-date nature, as it represents the most recent meta-analysis examining the prognostic significance of Gal-9 across various cancer subtypes since the last analysis of this kind in 2018. Additionally, we included hematological cancers, which had not been previously reported. Our study encompassed patients from diverse regions, including Asia, Europe, North America, and Africa, thereby introducing greater variability in ethnicities. Furthermore, a wide range of Gal-9 detection methods were included in our study. Compared to older publications, which primarily employed IHC or IF staining on tumor blocks for Gal-9 detection, an increasing number of recent publications have begun measuring circulating Gal-9 levels using plasma samples from patients. In hematological cancers, all measurements were obtained from plasma using either ELISA or qPCR on B lymphocytes isolated from peripheral blood for Gal-9 mRNA quantification.

Despite the comprehensiveness of this study, it is limited by several factors. First, there is intrinsic heterogeneity among the cancer types examined. Histological subtypes, such as small cell and non-small cell lung carcinoma, exhibit different clinical behaviors, prognoses, and treatment strategies. Long-term clinical outcomes are influenced by multiple confounding factors, including age groups, treatment received, performance status, and stage of disease at diagnosis, which were not necessarily balanced or matched between the high and low Gal-9 expression groups. Some studies did not report the follow-up time, making it difficult to determine whether the study duration was sufficiently long for events such as recurrence or death to occur, particularly in slow-growing cancers.

Second, unlike some commonly used biomarkers such as HER2 [58] or PD-L1 [59], there is no universal standardized cutoff value for defining high and low Gal-9 expression in histological specimens. Significant variability in Gal-9 cutoff values has been observed across different studies. The absence of a unified reporting system may introduce subjectivity in interpreting Gal-9 expression. Most studies have relied on the median level of expression as the cutoff for classifying patients as “high” or “low” expressors of Gal-9. We strongly advocate establishing a well-defined cutoff value for Gal-9 in future clinical studies, considering the expression in both tumor and immune cells (combined positive score), as this is more clinically and mechanistically relevant. Additionally, serum Gal-9 expression has been reported to be influenced by medical conditions, such as autoimmune conditions and infections [60]. A recent study also showed that serum Gal-9 levels in patients with lung cancer are significantly lower in smokers than in non-smokers [61]. It is imperative to consider potential confounding concomitant physiological conditions when interpreting patients’ serum Gal-9 levels.

Finally, negative studies may be underreported, which could potentially exaggerate the relationship between Gal-9 expression and improved prognosis. Although we have utilized three major databases namely PubMed, Embase, and Web of Science, some non-English literature may remain uncaptured. The interpretation of the prognostic value of Gal-9 in hematological cancers is further hindered by the inclusion of only three studies, all of which focused on the same subtypes.

In summary, our study provides the most up-to-date information on the prognostic value of Gal-9 in different cancer subtypes, particularly highlighting the potential different prognostic significance of extracellular Gal-9 due to its physiological function. Given that Gal-9 is increasingly recognized as an important target or immune checkpoint in the TME in the era of immunotherapy, further studies investigating the expression of extracellular Gal-9 and its association with cancer prognosis, using methods such as ELISA, would help elucidate this paradoxical observation.

## Supporting information

S1 Fig Funnel Plot of OS/CSS studies in solid tumors.
(TIF)

S2 Fig Funnel Plot of DFS/RFS studies in solid tumors.
(TIF)

S3 Fig Funnel Plot of PFS/TTT studies in hematological cancers.
(TIF)

S4 Fig Sensitivity analysis for Gal-9 expression with OS/CSS in solid tumors.
(TIF)

S5 Fig Sensitivity analysis for Gal-9 expression with DFS/RFS in solid tumors.
(TIF)

S6 Fig Sensitivity analysis for Gal-9 expression with PFS/TTT in hematological cancers.
(TIF)

S1 File Summary of all the studies identified in literature search.
(XLSX)

S2 File PRISMA checklist.
(DOCX)

S1 Table Table listing the outcomes and Gal-9 localization of the studies included.
(DOCX)
