# Supplementary figures and images for: New insights on Galectin-9 expression in cancer prognosis: An updated systemic review and meta-analysis
Source: PLoS One. 2025 Mar 26;20(3):e0320441. doi: 10.1371/journal.pone.0320441 (PMC11940609; doi:10.1371/journal.pone.0320441)

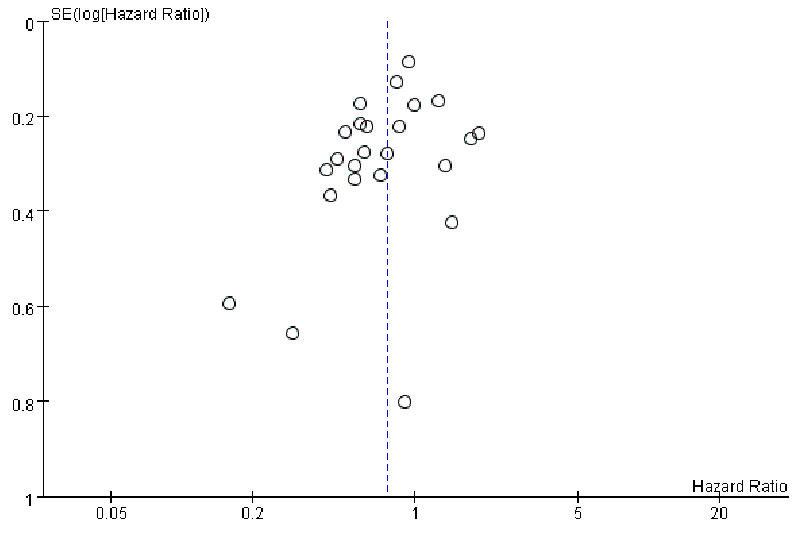

Supplement: S1 Fig — (TIF) [file pone.0320441.s001.tif]

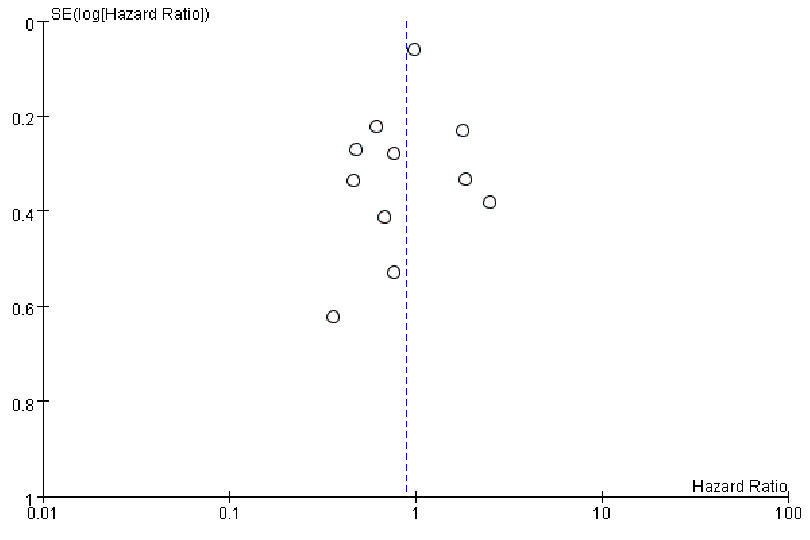

Supplement: S2 Fig — (TIF) [file pone.0320441.s002.tif]

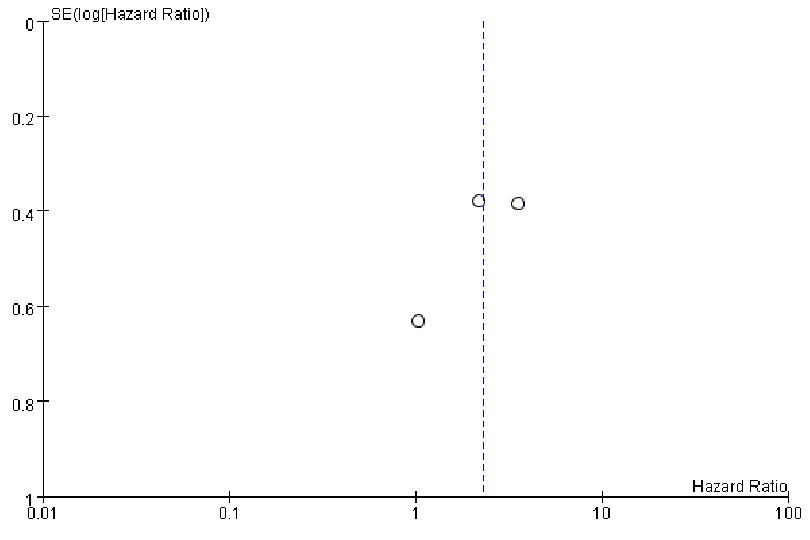

Supplement: S3 Fig — (TIF) [file pone.0320441.s003.tif]

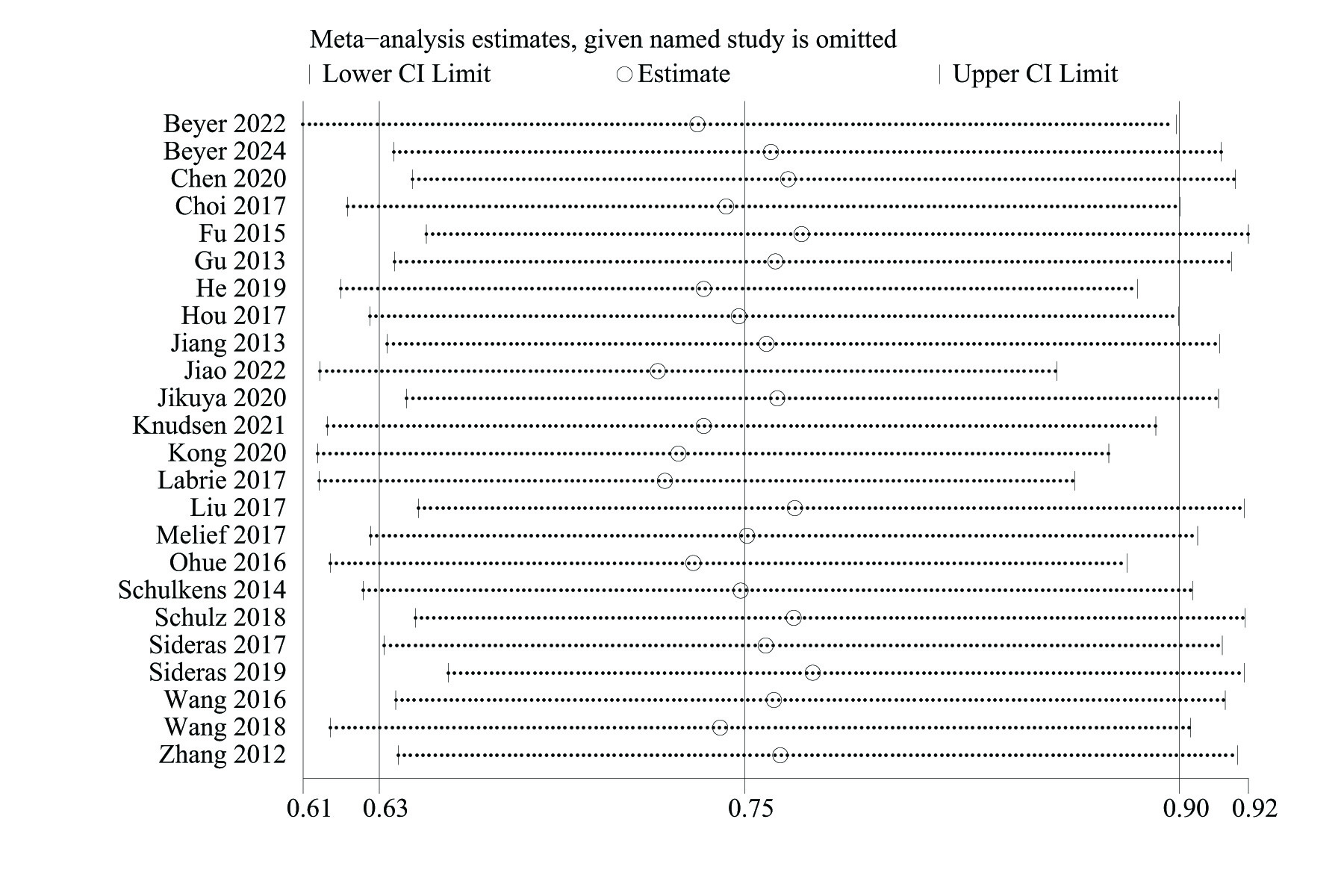

Supplement: S4 Fig — (TIF) [file pone.0320441.s004.tif]

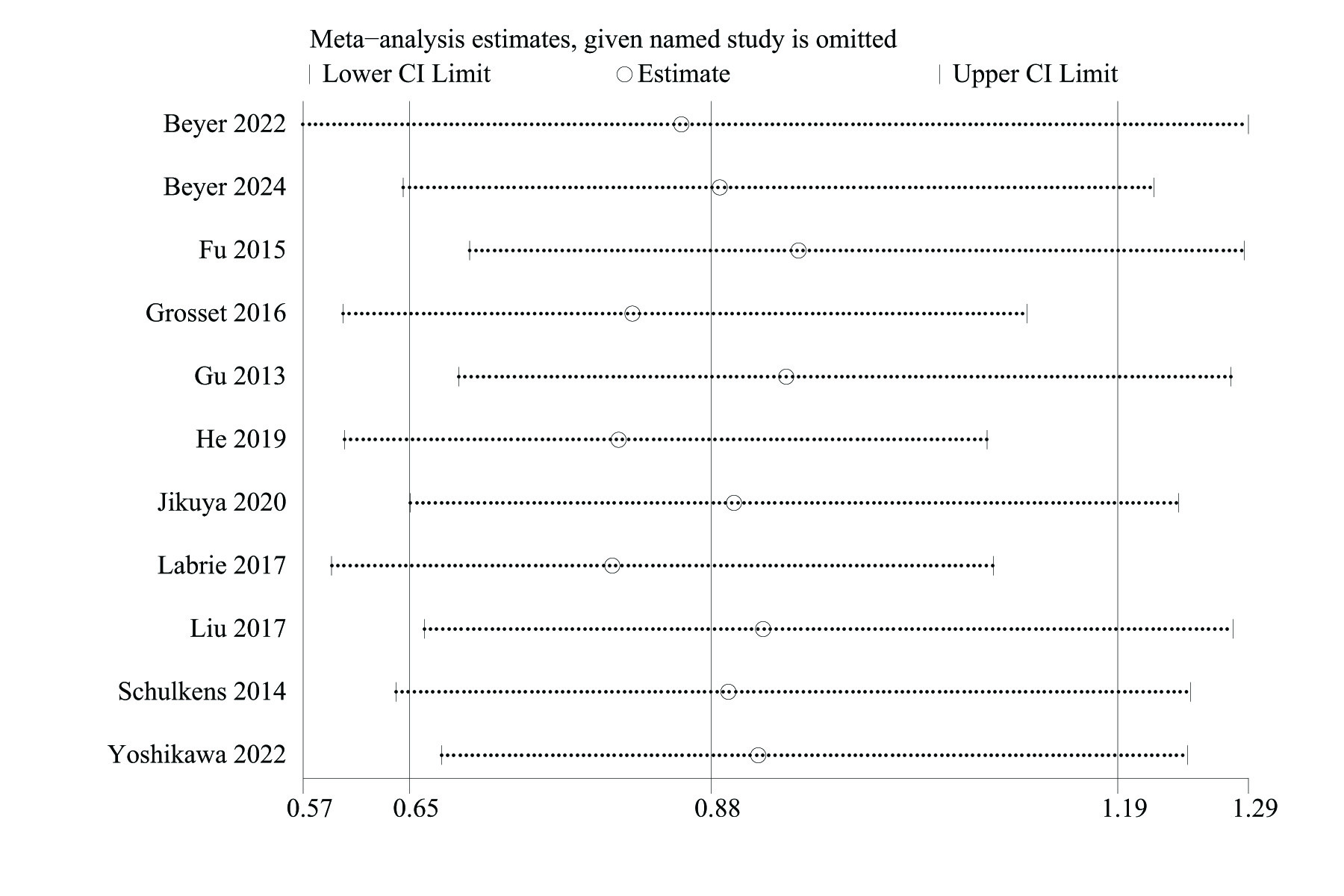

Supplement: S5 Fig — (TIF) [file pone.0320441.s005.tif]

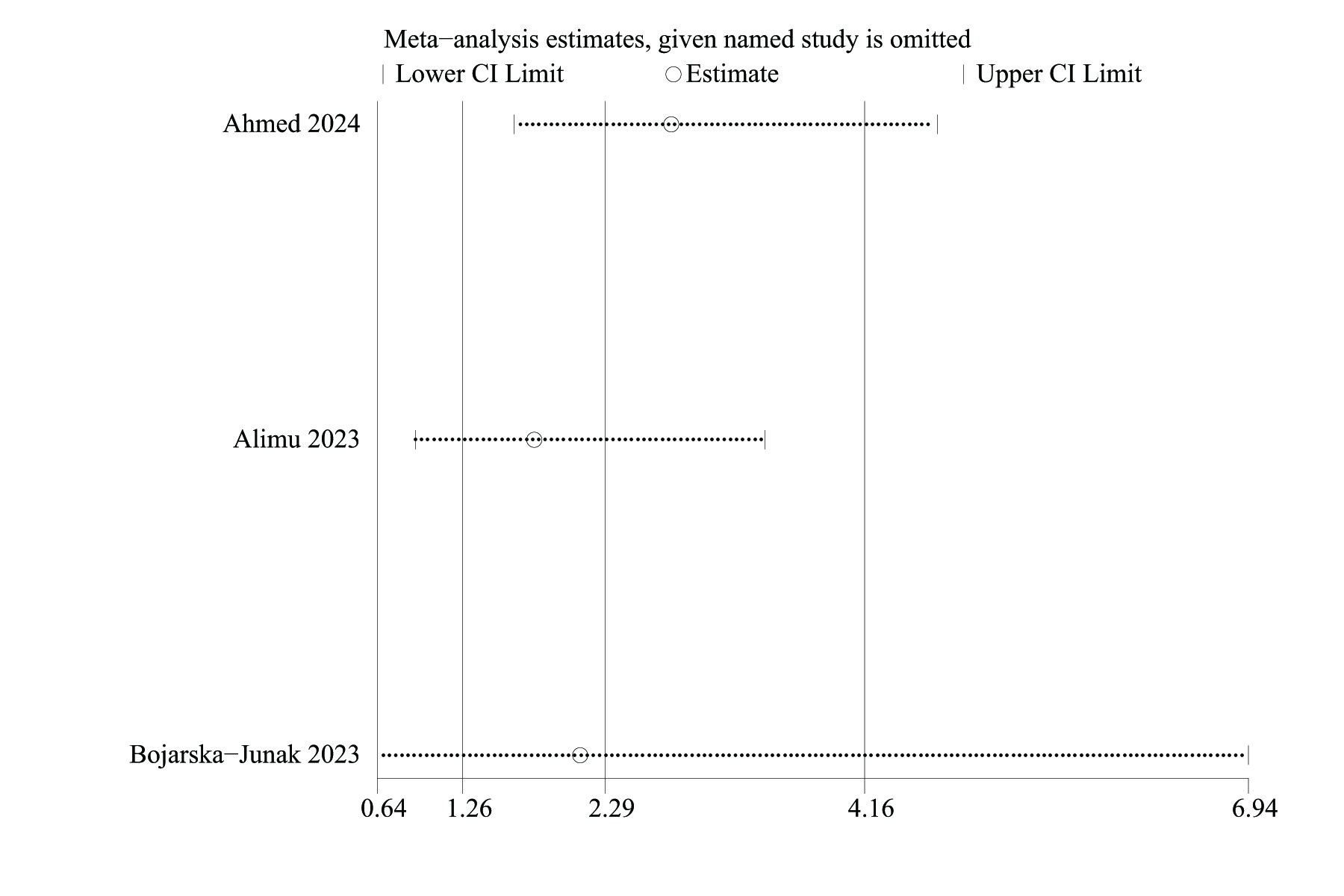

Supplement: S6 Fig — (TIF) [file pone.0320441.s006.tif]
