# Supplementary material for: New insights on Galectin-9 expression in cancer prognosis: An updated systemic review and meta-analysis
Source: PLoS One. 2025 Mar 26;20(3):e0320441. doi: 10.1371/journal.pone.0320441 (PMC11940609; doi:10.1371/journal.pone.0320441)
Supplement: S1 Table — (DOCX) [file pone.0320441.s009.docx]

| **Author** | **HR for OS/CSS (95%CI)** | **p-value** | **HR for RFS/DFS (95%CI)** | **p-value** | **Gal-9 localization**  **(I/E)** |
| --- | --- | --- | --- | --- | --- |
| **Solid tumors** |  |  |  |  |  |
| Knudsen | 0.99 (0.7-1.4) | 1 |  |  | I |
| Yoshikawa |  |  | 0.365(0.108-1.236) | 0.106 | I |
| Melief | 0.712 (0.377–1.347) | 0.297 |  |  | I |
| Wang 2016 | 0.549 (0.303–0.995) | 0.048 |  |  | I |
| Choi | 0.85 (0.55-1.31) | 0.467 |  |  | I |
| Hou | 0.905 (0.189-4.322) | 0.9 |  |  | I |
| Jiang | 0.60 (0.35-1.01) | 0.056 |  |  | I |
| Wang 2018 | 0.830 (0.643-1.072) | 0.153 |  |  | I |
| Fu | 0.418 (0.226-0.772) | 0.005 | 0.477 (0.281-0.810) | 0.006 | I |
| Jikuya | 0.300 (0.083-1.086) | 0.067 | 0.680 (0.304-1.520) | 0.347 | I |
| Liu | 0.46 (0.26-0.81) | 0.008 | 0.62 (0.40-0.95) | 0.03 | I |
| Gu | 0.58 (0.38-0.88) | 0.011 | 0.463 (0.24-0.89) | 0.023 | I |
| Jiao | 1.877 (1.179-2.744) | 0.004 |  |  | I |
| Kong | 1.25 (0.90-1.73) | 0.187 |  |  | I |
| Sideras 2017 | 0.62 (0.40-0.97) | 0.038 |  |  | I |
| Sideras 2019 | 0.16 (0.05-0.47) | 0.001 |  |  | E |
| Zhang | 0.578 (0.410-0.820) | 0.002 |  |  | I |
| Chen | 0.436 (0.212, 0.897) | 0.024 |  |  | I |
| He | 1.439 (0.627-3.303) | 0.391 | 2.482 (1.178-5.227) | 0.017 | I |
| Schulkens | 0.76 (0.44-1.33 | 0.333 | 0.76 (0.44-1.31) | 0.336 | I |
| Beyer 2022 | 0.930 (0.783, 1.105) | 0.41 | 0.980 (0.869, 1.105) | 0.741 | I |
| Labrie | 1.734 (1.066, 2.822) | 0.027 | 1.795 (1.140, 2.825) | 0.012 | E |
| Schulz | 0.503 (0.318, 0.796) | 0.003 |  |  | I |
| Beyer 2024 | 0.55 (0.286, 1.059) | 0.074 | 0.762 (0.271, 2.143) | 0.606 | I |
| Grosset |  |  | 1.84 (0.96-3.54) | 0.121 | I |
| Ohue | 1.34 (0.72-2.46) | 0.35 |  |  | I |
| **Hematological cancers** | **HR for PFS (95%CI)** | | | | **Gal-9 localization** |
| Ahmed | 1.03(0.30-3.52) | | | | E |
| Alimu | 3.53 (1.66-8.95) | | | | E |
| Bojarska-Junak | 2.19 (1.046-4.310) | | | | E |

I: Intracellular

E: Extracellular
